# Variables associated with cortical motor mapping thresholds: A retrospective data review with a unique case of interlimb motor facilitation

**DOI:** 10.3389/fneur.2023.1150670

**Published:** 2023-04-11

**Authors:** Yinchen Song, James V. Surgenor, Zachary T. Leeds, John H. Kanter, Pablo Martinez-Camblor, William J. Smith, M. Dustin Boone, Alexander T. Abess, Linton T. Evans, Erik J. Kobylarz

**Affiliations:** ^1^Department of Neurology, Dartmouth-Hitchcock Medical Center, Lebanon, NH, United States; ^2^Geisel School of Medicine, Dartmouth College, Hanover, NH, United States; ^3^Haverford College, Haverford, PA, United States; ^4^Section of Neurosurgery, Dartmouth-Hitchcock Medical Center, Lebanon, NH, United States; ^5^Department of Anesthesiology, Dartmouth-Hitchcock Medical Center, Lebanon, NH, United States; ^6^Department of Biomedical Data Science, Geisel School of Medicine, Dartmouth College, Hanover, NH, United States; ^7^Thayer School of Engineering, Dartmouth College, Hanover, NH, United States

**Keywords:** brain tumor, intraoperative neuromonitoring, motor mapping, stimulation induced seizures, facilitation

## Abstract

**Introduction:**

Intraoperative neuromonitoring (IONM) is crucial to preserve eloquent neurological functions during brain tumor resections. We observed a rare interlimb cortical motor facilitation phenomenon in a patient with recurrent high-grade glioma undergoing craniotomy for tumor resection; the patient’s upper arm motor evoked potentials (MEPs) increased in amplitude significantly (up to 44.52 times larger, *p* < 0.001) following stimulation of the ipsilateral posterior tibial nerve at 2.79 Hz. With the facilitation effect, the cortical MEP stimulation threshold was reduced by 6 mA to maintain appropriate continuous motor monitoring. It likely has the benefit of reducing the occurrence of stimulation-induced seizures and other adverse events associated with excessive stimulation.

**Methods:**

We conducted a retrospective data review including 120 patients who underwent brain tumor resection with IONM at our center from 2018 to 2022. A broad range of variables collected pre-and intraoperatively were reviewed. The review aimed to determine: (1) whether we overlooked this facilitation phenomenon in the past, (2) whether this unique finding is related to any specific demographic information, clinical presentation, stimulation parameter (s) or anesthesia management, and (3) whether it is necessary to develop new techniques (such as facilitation methods) to reduce cortical stimulation intensity during intraoperative functional mapping.

**Results:**

There is no evidence suggesting that clinical presentation, stimulation configuration, or intraoperative anesthesia management of the patient with the facilitation effect were significantly different from our general patient cohort. Even though we did not identify the same facilitation effect in any of these patients, we were able to determine that stimulation thresholds for motor mapping are significantly associated with the location of stimulation (*p* = 0.003) and the burst suppression ratio (BSR) (*p* < 0.001). Stimulation-induced seizures, although infrequent (4.05%), could occur unexpectedly even when the BSR was 70%.

**Discussion:**

We postulated that functional reorganization and neuronal hyperexcitability induced by glioma progression and repeated surgeries were probable underlying mechanisms of the interlimb facilitation phenomenon. Our retrospective review also provided a practical guide to cortical motor mapping in brain tumor patients under general anesthesia. We also underscored the need for developing new techniques to reduce the stimulation intensity and, hence, seizure occurrence.

## 1. Introduction

The routine use of intraoperative neuromonitoring (IONM) during intracranial tumor resection has allowed for maximal surgical resection while ensuring preservation of cortical and subcortical structures in eloquent areas of the brain (1–3). In particular, functional mapping and monitoring based on short-latency somatosensory evoked potential (SSEP) and motor evoked potential (MEP) techniques are useful for evaluation and protection of sensorimotor pathways during resections of peri-Rolandic tumors.

We recently observed an interlimb motor facilitation phenomenon in a patient undergoing resection of a recurrent high-grade glioma. The tumor was located on the right side in the medial superior frontal region anterior to the primary motor cortex. The facilitation effect induced a significant increase in cortical MEP (cMEP) amplitudes in the left upper extremity muscles when directly stimulating the primary motor cortex posterior to the glioma. Interestingly, the facilitation effect was only present whenever the left-side posterior tibial nerve (PTN) at the ankle was stimulated for SSEP monitoring immediately before cMEP stimulation. Left-side median or ulnar nerve stimulation did not induce any facilitation effect. With the facilitation effect, the stimulation current for eliciting cMEPs was reduced from 17 mA to 11 mA. Such a facilitation effect is especially intriguing in that it can decrease the cortical stimulation intensity drastically and potentially reduce the occurrence of stimulation-induced seizures (4), inaccurate mapping due to current spread (5), and/or tissue damage (6).

MEP facilitation has been utilized as a technique to optimize MEP monitoring with less trial-to-trial variability in spinal surgeries when patients have significant iatrogenic neuromuscular block on board or compromised excitability of different parts of the motor pathway (7) (i.e., motor cortex or anterior horn cells). Several prior studies have described successful interlimb facilitation (i.e., lower extremity peripheral nerve stimulation facilitating upper extremity MEPs) in patients undergoing spinal surgeries; however, these studies utilized tetanic stimulation of peripheral nerves at a much higher frequency (i.e., 50 Hz) for 5 s with a fixed interstimulation interval prior to transcranial MEP stimulation (7–9). The underlying physiological mechanisms responsible for the modulatory effects of afferent electrical stimulation on the excitability of cortical, subcortical and spinal circuits have yet to be determined. Not surprisingly, there are few reported studies evaluating the utility of facilitation in cortical motor mapping during brain tumor resections. To date, we are aware of only one published study reporting post-tetanic motor facilitation during pediatric craniotomy cases (10).

In this manuscript, we present a retrospective review of patients with peri-Rolandic tumors who underwent craniotomy for tumor resection with IONM under general anesthesia over the past 4 years at Dartmouth-Hitchcock Medical Center (DHMC), a rural academic medical center in northern New England. One of the major goals of this study was to determine whether this unique facilitation effect was present in other patients. We also aimed to elucidate the underlying mechanism (s) of the aforementioned interlimb motor facilitation phenomenon. In addition, the stimulation threshold in the patient with interlimb facilitation seemed to be relatively high (i.e., 17 mA). We further analyzed the data collected from our patient cohort to determine whether this specific case was an outlier with respect to the patient’s clinical representation, stimulation configuration, or intraoperative anesthesia management. We anticipated identifying variables that may have contributed to the high stimulation threshold utilized in this patient, and providing guidance on parameter selection and optimization for intraoperative cortical motor mapping in the future. Finally, we expect to gain some insight into the need to develop facilitation methods for cortical motor mapping. Do we truly need some new “tricks” to reduce the occurrence of stimulation-induced seizures?

## 2. Materials and methods

### 2.1. Patient selection

**Figure 1:**
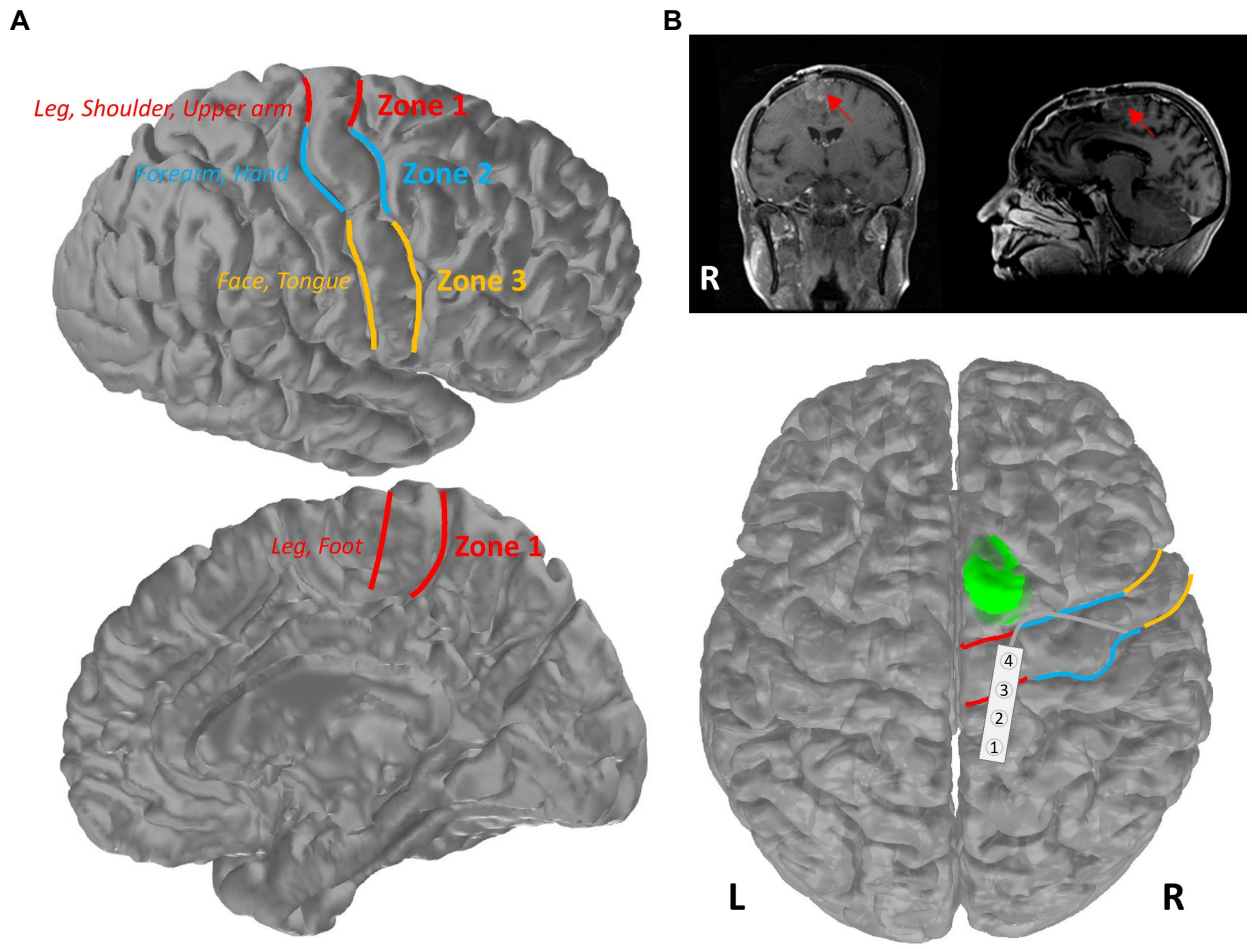
Primary motor cortex of the brain. **(A)** Schematic of Zones 1–3 in the primary motor cortex. Patients were separated into three groups based on the mapping location within these three zones (red: Zone 1; blue: Zone 2; yellow: Zone 3). **(B)** Illustration of the tumor location of the patient in the highlighted case. Patient’s pre-operative MR images were presented in coronal and sagittal views, with a red arrow pointing toward the tumor. The tumor is shown in green on an averaged brain atlas surface. Zones 1–3 were delineated with the corresponding colors as shown in **(A)**. The strip electrode used for functional mapping was placed over the primary motor and sensory areas in Zone 1.

The research protocol for this retrospective review study was approved by the Institutional Review Board (IRB) at DHMC. Patients who underwent surgical resection of brain tumors with IONM between 2018 and 2021 at DHMC were selected for review of their electronic medical records (EMRs) and IONM data in this retrospective study. Patients who underwent awake craniotomy for language and/or motor mapping were excluded from further analyses, as anesthesia may play an important role in determining cortical stimulation thresholds. In accordance with Magill et al.’s classification (11), we could further divide the cohort into three groups based on the tumor and motor mapping locations along the motor cortex (Figure 1A), where Zone 1 covers the medial portion of the motor cortex representing motor function of the lower extremity, trunk, shoulder and upper arm; Zone 2 (lateral to Zone 1) is responsible for forearm and hand motor functions; and Zone 3 (inferior to Zone 2) encompasses the motor functions for facial and tongue muscles.

### 2.2. Data review

The review was performed on data collected from patients undergoing tumor resection with motor mapping under general anesthesia. Detailed methodological information regarding the surgical procedure, anesthesia management, and IONM can be found in Supplementary material. The primary goal of this retrospective review study was to explore the underlying mechanism (s) of the interlimb facilitation phenomenon and to assess whether it has been overlooked in the past. IONM data were reviewed for the presence of any documented changes in cMEPs during the intraoperative continuous monitoring period. The changes, if any, were reviewed further in original recordings to determine whether they may be attributed to the abovementioned facilitation effects. Second, the review aimed to explore what variables may be associated with the high stimulation threshold (i.e., 17 mA) reported in the highlighted case. We aimed to elucidate whether the reported facilitation effect could be caused by any aberrant clinical variables or stimulation setups. With the data collected from our patient cohort, we intended to establish a model that can justify whether the stimulation threshold in the highlighted case was within the normal range. Finally, we aimed to determine the occurrence of stimulation-induced seizures in this patient cohort.

Specifically, data for variables such as the stimulation intensity and corresponding parameters (i.e., pulse duration, frequency, polarity, etc.), the presence of intraoperative seizures triggered by cortical stimulation, and the stimulation location were collected for further analyses. To have a fair comparison of the stimulation thresholds, the stimulation current intensity was normalized based on the pulse duration (i.e., 500 μs) using the equation for calculating the charge density (12). For example, for a stimulation with a pulse duration of 300 μs, the stimulation current intensity was adjusted by multiplying by a factor of 3/5. The stimulation location was approximated based on the IONM report and intraoperative imaging and classified into three different “zones” based on Magill et al.’s definitions (11). The burst suppression ratio (BSR, in %), defined as the ratio of the duration of suppressed electrocorticography (ECoG) signals to the studied time interval, was determined within 1-min ECoG segments before and after the time of cortical motor mapping and rounded up to the nearest multiple of 10. In addition to typical demographic information, such as age, sex and weight, patients’ EMRs were reviewed to determine the laterality of the tumor (labeled as “laterality”), the pathological findings of the tumor (labeled as “tumor”), whether the patient had a prior resection in nearby regions (labeled as “recurrent”) and operating/anesthesia duration before the motor mapping (labeled as “surgical duration”). Anesthesia records were reviewed to extract the infusion rate of propofol, opioid (i.e., remifentanil), and other adjunct anesthetics (i.e., ketamine and/or dexmedetomidine), as well as the mean arterial pressure (MAP, in mmHg) before and after the time of cortical motor mapping. The MAP was also averaged within the 2-min window and rounded up to the nearest multiple of 10.

### 2.3. Statistical analysis

Statistical analyses were performed in R, a programming language for statistical computing and graphics. Of note, data from the highlighted case were not included in the following analyses unless specified. Demographic and clinical data were analyzed using descriptive statistics. For each variable, the Wilcoxon signed-rank test and one-way analysis of variance (ANOVA) were used to compare the stimulation current thresholds. Some quantitative variables, such as the propofol infusion rate, MAP, and BSR, were grouped into 3–4 bins to facilitate the univariate statistical comparison. Bonferroni correction was used for multiple-comparison correction. A multivariate linear regression model was adopted to identify the effects (i.e., β) of variables that can determine the stimulation current intensity for cortical motor mapping. Interactions among variables were also included in the model. A scatterplot matrix was inspected to ensure that quantitative variables were not correlated with each other. The best-performing model based on the Akaike information criterion was selected *via* a forward stepwise algorithm. Data from the highlighted case were implemented in this selected model to estimate the range of stimulation current intensity used and therefore to infer whether the actual stimulation current threshold used for the patient was an outlier.

### 2.4. Data availability statement

We are not able to make the patient’s data publicly available according to our current IRB protocol. The IONM protocols, stimulation parameters and data analysis code are available on request to the corresponding author.

## 3. Results

**Figure 2:**
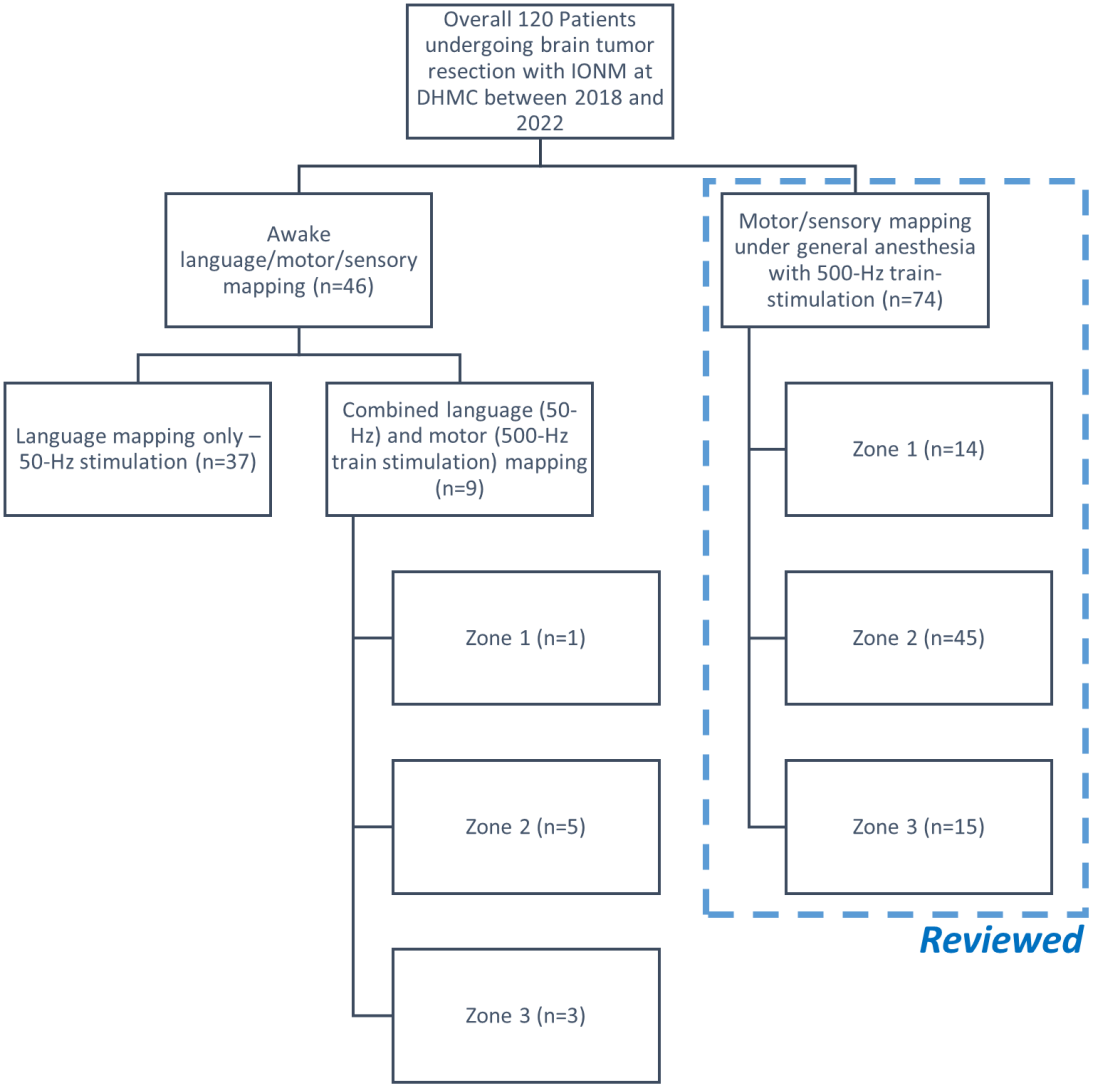
Flow chart of the retrospective cohort study. The patient in the highlighted case, which was not included in this chart, underwent motor mapping in Zone 1 under general anesthesia. Only the cases performed under general anesthesia were included for further review.

A total of 120 patients, in addition to the patient in the highlighted case, underwent brain tumor resection with IONM at DHMC over the last 4 years (Figure 2). There were 46 patients who underwent awake craniotomy for tumor resection near the eloquent regions related to language functions. Nine out of these 46 patients also underwent motor mapping either under light sedation or awake conditions. On the other hand, 74/120 patients underwent tumor resection with intraoperative cortical motor mapping under general anesthesia without inhalational anesthetics. Data from these 74 patients were acquired for further analyses to compare with those of the patient in the highlighted case.

### 3.1. Highlighted case

A 57-year-old right-handed female diagnosed with a recurrent anaplastic astrocytoma with progression to glioblastoma (WHO grade IV) presented for a right-side craniotomy for tumor resection.

She initially presented following a generalized tonic–clonic seizure. A magnetic resonance imaging (MRI) scan revealed a right-side non-enhancing frontal lesion (9 × 11 × 13 mm^3^). She underwent subtotal resection at that time followed by adjuvant radiation and temozolomide. Pathology was consistent with an anaplastic glial neoplasm (WHO grade III). Her chemotherapy was complicated by thrombocytopenia. She had a breakthrough seizure and developed a recurrent tumor a year later. She underwent a second craniotomy with resection of an additional viable tumor followed by implantation of FDA-approved carmustine wafers (Gliadel^®^, Arbor Pharmaceutical, Atlanta, GA). This procedure was also followed by a nine-month course of Optune^®^ tumor treating field (TTF) therapy.

Her MRI findings 2 years after initial surgery were concerning for tumor progression as well as possible transformation. She was taking levitiracitam (1,000 mg twice a day) and lamotrigine (250 mg twice a day) for seizure control. She had experienced right hand tremor and head-nodding spells without loss of consciousness. She did have an episode when she had some trouble speaking, without loss of consciousness. Her preoperative neurologic exam did not reveal any concerns for her motor and sensory functions. Spontaneous language was fluent and appropriate. Naming and repetition were intact. She underwent a third craniotomy to remove the recurrent tumor. IONM was utilized to continuously monitor the motor pathways, including the primary motor cortex and the corticospinal tract (CST), during this craniotomy.

**Figure 3:**
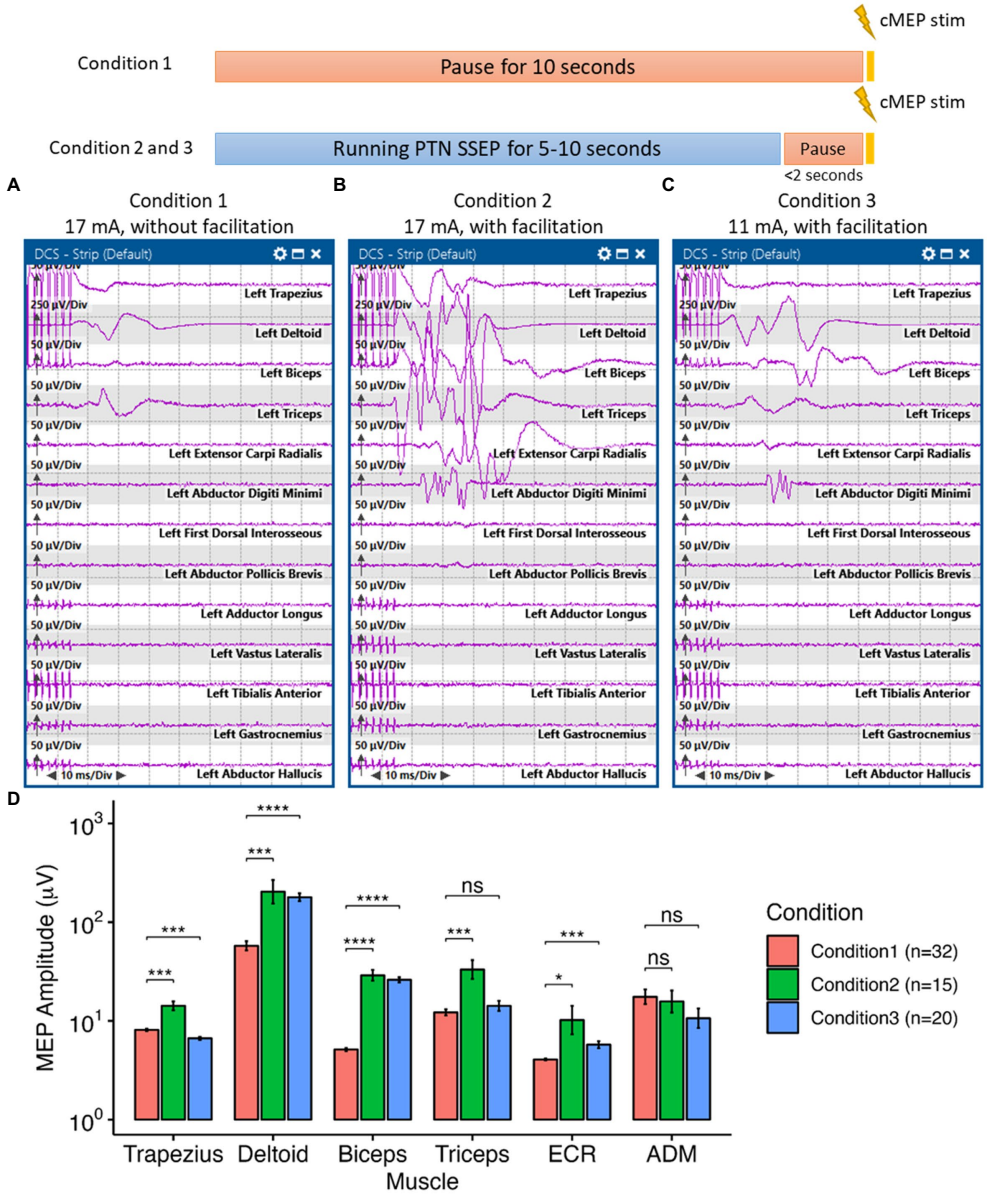
Interlimb motor facilitation in the highlighted case. Here, all cortical motor stimulation was delivered with a pulse duration of 300 μs at 500 Hz every 10 s. Under Condition 1, no other electrical stimulation was delivered to the patient during the 10-s interval. Under condition 2 and 3, posterior tibial nerve (PTN) somatosensory evoked potential (SSEP) was monitored during the 10-s interval, which induced the “facilitation” effect. **(A)** Without PTN facilitation, we were able to elicit small motor responses on the contralateral shoulder and upper arm by stimulating the cortical surface at 17 mA. **(B)** With the facilitation effect induced by stimulating the PTN, we were able to obtain greater responses, compared to previous responses, with more complex morphologies on the previously mentioned muscles as well as the forearm and hand muscles by stimulating the same cortical area at 17 mA. **(C)** The threshold for eliciting contralateral shoulder, arm and hand cortical motor evoked potentials (cMEPs) was reduced to 11 mA with the facilitation effect. **(D)** The facilitation phenomenon was reproducible and had a significant effect in increasing the amplitudes of the cMEPs in the shoulder, arm and hand muscles. In the ANOVA test to compare the cMEP amplitudes under different conditions, the *p-*value was less than 0.001. In the pairwise comparison of cMEP amplitudes between conditions for each muscle, a *p-*value less than 0.05, with Bonferroni correction, was considered significant. ECR: extensor carpi radialis, ADM, abductor digiti minimi. Significance level: ns, not significant, *p < 0.05, **p < 0.01, ***p < 0.001, ****p < 0.0001.

During intraoperative functional mapping, a 1 × 4 strip electrode was placed over the medial frontoparietal cortical surface by the surgeon, with contact 1 positioned posteriorly and contact 4 positioned anteriorly (Figure 1B). We were able to obtain a sensory response from contacts 1 and 2 by stimulating the PTN. Contacts 3 and 4 appeared to be on top of the primary motor cortex. However, we could not use the typical phase reversal technique (13–16) to determine the locations of sensory and motor cortex in this particular area (see Supplementary material). All contacts were stimulated to determine the motor cortex location. We were only able to acquire cMEPs from the left-side deltoid (DEL), triceps (TRI), and extensor carpi radialis (ECR) muscles when stimulating contact 4 cathodally at 17 mA (interstimulus interval, ISI = 2 ms, pulse width = 300 μs, and pulse count = 7; Figure 3A). A ball-tip monopolar probe (2.3 mm, Medtronic Xomed, Jacksonville, FL) was also utilized to map the motor areas. We were able to obtain the same motor responses adjacent to contact 4 by stimulating the ball-tip probe at 15 mA. When the ball-tip probe was moved laterally to contact 4, we were able to obtain hand motor responses at 10 mA. Using the ball-tip probe, cortical areas anterior and posterior to contact 4, as well as the tumor itself, were stimulated up to 18 mA without triggering any cMEPs. Therefore, we were confident that contact 4 was indeed on top of the primary motor cortex.

During tumor resection, cMEPs were continuously monitored with a fixed 10-s interval. Upper and lower limb SSEPs were obtained intermittently during the 10-s interval every 5–10 min. To avoid SSEP-induced movement convolving with cMEPs, we manually paused the SSEP stimulation a couple seconds before eliciting cMEPs. We noticed a significant increase in the amplitude of cMEPs (i.e., up to 44.52 times larger in the cMEP amplitudes of the upper arm under Condition 2 than those in Condition 1, *p* < 0.001, ANOVA, Figure 3D) whenever SSEPs were running ahead of the cortical motor stimulation (i.e., Condition 2 in Figure 3B). Only PTN stimulation enhanced the cMEPs of the upper arm (Figures 3B,C). Ulnar and median nerve stimulations, either individually or combined, did not have any augmentation effect on cMEPs. The stimulation threshold for triggering cMEPs could be reduced from 17 mA to 11 mA when PTN was stimulated ahead of time while still maintaining equivalent, if not better, cMEPs in those upper extremity muscles (Figure 3C). This “facilitation” phenomenon was reproducible (Figure 3D) and did not require stringent timing of sequential peripheral and cortical stimulations. Peripheral stimulation of the PTN lasted between 4 and 10 s before cortical stimulation. The average interval between peripheral and cortical stimulations was approximately 462.83 ± 263.22 ms, with a range of 128.9 to 1009.3 ms. The patient’s anesthetic and hemodynamic management remained stable and unchanged during the functional mapping and monitoring periods.

### 3.2. Changes in cMEP amplitudes

Decrements in cMEP amplitudes were documented in 48 out of 74 patients (64.86%) with motor mapping under general anesthesia, which is contrary to the facilitation effect we observed in the highlighted case. These decrements were found to be associated with the brain shift and the movement of the subdural strip electrodes, which were transient in nature and reversible if the surgeons were able to reposition the subdural strip electrodes on the original location. However, there were three patients for whom we failed to reposition the electrodes on the original location due to a significant brain shift during tumor resection. For a handful of patients (*n* = 5) we observed some transient increases in cMEPs during tumor resection, which was thought to be related to the subtle retraction applied to the resection cavity. These increases occurred randomly and did not reproduce consistently.

There were some interesting effects on the cMEPs from PTN stimulation documented in a 69-year-old female patient with a known history of non-small cell lung cancer who presented with a metastatic carcinoma in the right frontal parafalcine area. This patient did not have any prior brain tumor resection. We were able to map the lower extremity motor area at 11 mA. The amplitude of the cMEPs on the abductor hallucis brevis muscle increased proportionally when the PTN stimulation intensity was gradually increased from 20 to 40 mA (Supplementary Figure 1). However, the “facilitation” effect was not reliable and only presented for a few trials. Therefore, we do not believe this patient experienced the same facilitation mechanism as the one experienced by the patient in the highlighted case. Nevertheless, we did not see any other reports of consistent and reproducible increases in cMEP amplitude subsequent to peripheral nerve stimulation in the limbs.

### 3.3. Stimulation-induced seizures

Among 74 motor mapping cases under general anesthesia, three patients (4.05%) with glioma (2 high-grade and 1 low-grade) had stimulation-induced seizures at 10.73 ± 3.36 mA with an average BSR of 60 ± 10%. The stimulation intensities that triggered seizures were not significantly different from motor mapping thresholds in these three patients (*p* = 0.10, paired *t-*test). Two of these patients had seizures during the mapping process, which showed ECoG changes immediately after cortical stimulation in Zone 3 (14.4 and 10 mA). The other patient had a seizure during the continuous motor monitoring part of the procedure, with a stimulation of 7.8 mA in Zone 2 and an interstimulation interval of 10 s. No after-discharge was observed from 500-Hz stimulation under general anesthesia in this patient cohort.

### 3.4. Variables associated with cMEP threshold

The demographic and clinical data of these 74 patients, as well as the patient in the highlighted case, are summarized in Table 1. Eleven out of 74 patients (14.86%) had at least one prior surgery to remove the brain tumor; however, they presented again for resection due to tumor regrowth. Among these 11 patients, two patients with glioma (WHO grade II astrocytoma and WHO grade III anaplastic oligodendroglioma) and one with metastatic carcinoma had two prior resections. The patient in the highlighted case also had two prior resections of the brain tumor. We do not have access to the IONM data from the prior surgeries for these patients.

**Table 1:** Summarized variables between the highlighted case and other reviewed cases.

Variable		Value	
		Reviewed cases	Highlighted case
Number of patients		74	1
Sex	Female	31 (41.89%)	Female
Age at operation (years)		62.25 ± 14.07	57
Weight (kg)		87.52 ± 23.56	62.9
BMI		29.38 ± 6.86	22.9
Tumor laterality	Right	43 (58.11%)	Right
Tumor pathology	Glioma	47 (63.51%)	Glioma
	Metastasis	25 (33.78%)	
	Meningioma	2 (2.70%)	
Prior resection	Yes	11 (14.86%)	Yes
	×1	8 (10.81%)	
	×2	3 (4.05%)	1
Anesthesia duration before mapping (min)		120.45 ± 40.43	175
Propofol infusion rate (mcg/kg/min)		126.00 ± 32.66	175
Burst suppression ratio (%)		55.46 ± 17.06	80
Median arterial pressure (mmHg)		81.85 ± 9.50	90
Number of stimulation sites	All	100	1
	Zone 1	24 (24%)	1
	Zone 2	57 (57%)	
	Zone 3	19 (19%)	
Stimulation current (mA)	All	8.28 ± 3.86	
	Zone 1	9.48 ± 4.02	17 (11*)
	Zone 2	7.13 ± 3.44	
	Zone 3	10.24 ± 3.82	
Normalized stimulation current (mA)	All	7.83 ± 3.68	
	Zone 1	9.26 ± 3.96	10.2 (6.6*)
	Zone 2	6.69 ± 3.21	
	Zone 3	9.44 ± 3.58	

Categorical data were summarized as the number of patients within the category followed by the percentage inside the parenthesis; quantitative data were summarized as the mean ± standard deviation.

* With facilitation effect.

**Figure 4:**
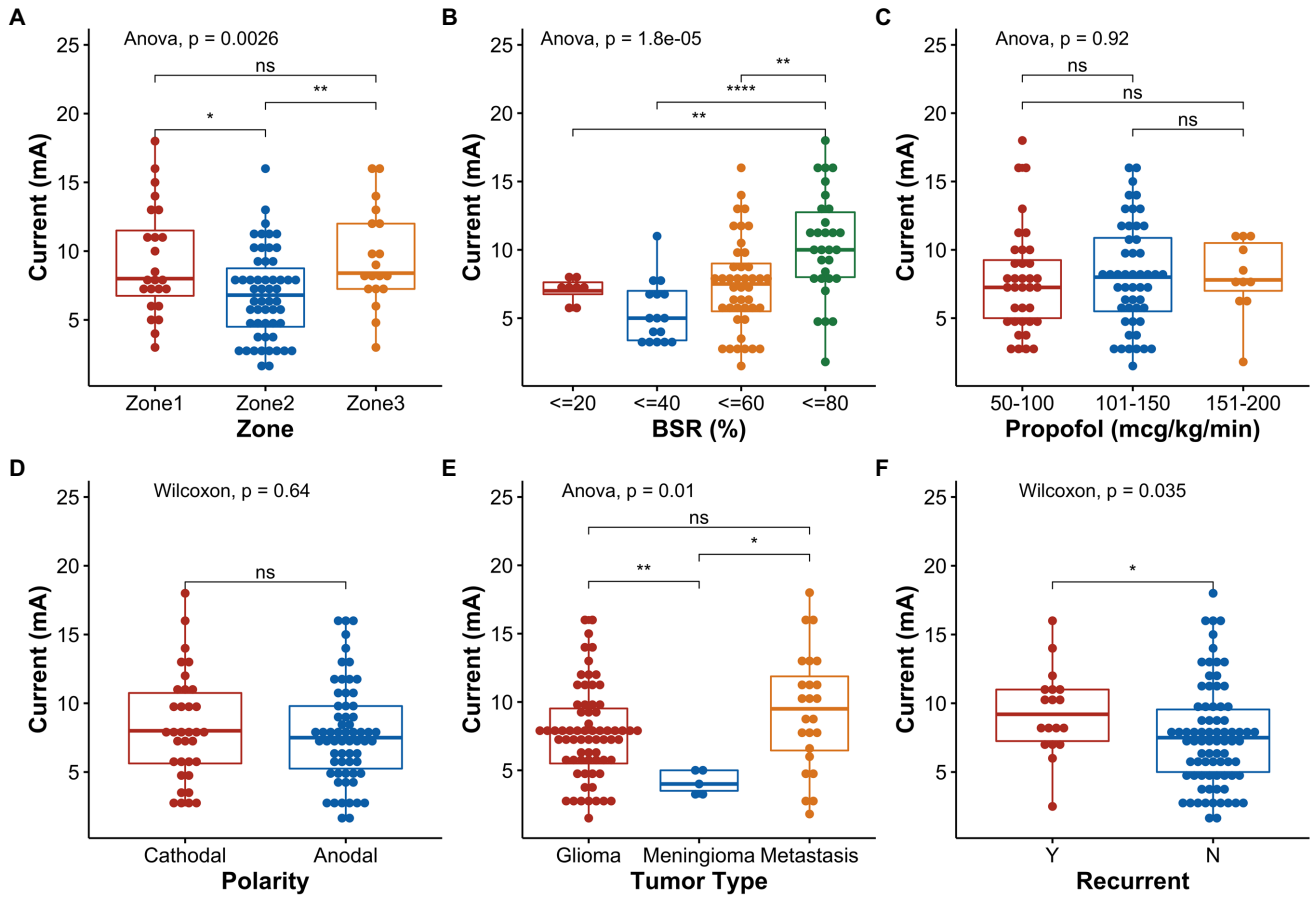
Boxplots and statistical comparisons of the normalized stimulation current thresholds for selected variables. **(A)** Stimulation zone, **(B)** burst suppression ratio (BSR), **(C)** propofol infusion rate, **(D)** stimulation polarity, **(E)** tumor type, and **(F)** tumor recurrence. Significance level: ns, not significant, *p < 0.05, **p < 0.01, ***p < 0.001, ****p < 0.0001.

For univariate comparison, the motor mapping threshold for Zone 2 (6.69 ± 3.21 mA) was significantly lower than those for Zone 1 (9.26 ± 3.96 mA) and Zone 3 (9.44 ± 3.58 mA) (*p* = 0.017 and *p* = 0.007, respectively, Figure 4A). A high BSR (60–80%) increased the overall stimulation threshold (Figure 4B). Prior resection significantly increased the stimulation threshold (*p* = 0.035, Figure 4F). Age, sex, weight, surgical duration before mapping, propofol infusion rate (Figure 4C), MAP, stimulation polarity (Figure 4D), and tumor type (i.e., glioma or metastasis) had no effect on the stimulation thresholds. In the few patients with motor mapping near the meningioma, it appeared that the stimulation threshold (4.10 ± 0.89 mA) was significantly lower than those obtained in patients with glioma and metastasis (7.74 ± 3.32 mA and 9.29 ± 4.33 mA, respectively, Figure 4E), regardless of the mapping zones.

**Figure 5:**
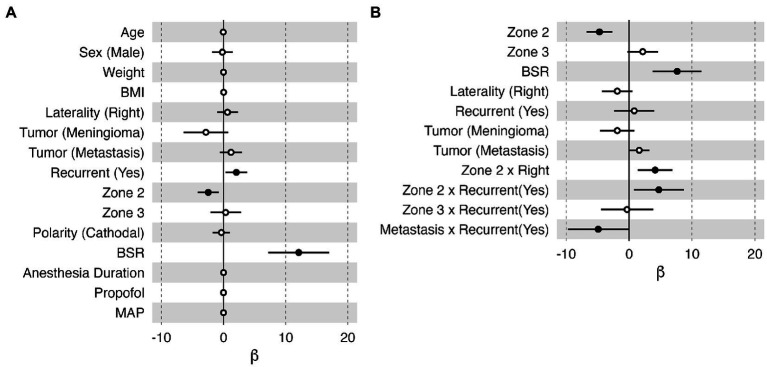
Bar plots of the effect (β) of each variable on the normalized stimulation current threshold in the multiple linear regression model. Each horizontal bar shows the 95% confidence interval of the effect of each variable. An effect with *p* < 0.05 was considered significant, illustrated with a filled black dot marking the mean value. **(A)** All the collected variables were included in this model. **(B)** The optimal model selected by the stepwise algorithm. Sex (female), laterality (left), tumor (glioma), recurrent (no), Zone 1, and polarity (anodal) were selected as references in this model; therefore, they do not have an estimation of β in these plots. BMI, body mass index; BSR, burst suppression ratio; MAP, mean arterial pressure.

In the linear regression model, age, sex, weight, BMI, anesthesia duration, propofol infusion rate, and MAP were found to have negligible effects on the stimulation threshold (i.e., β ≅ 0, Figure 5A); hence, they were not considered in the automatic model selection process. The final model (Figure 5B) reaffirmed that the BSR was the most influential variable that can positively affect the stimulation threshold [95% confidence interval of β: (3.74, 11.51), *p* = <0.001]. Zone 2 demonstrated a lower threshold than Zone 1 (*p* < 0.001); however, in this model, Zone 3 did not have any significantly different effects in comparison to Zone 1 (*p* = 0.088). When the tumor had a prior resection near Zone 2, the stimulation threshold was higher (*p* = 0.022), which may imply functional reorganization. Zone 2 on the right hemisphere also had a significantly higher threshold than the left side (*p* = 0.005) in general. Interestingly, the model indicated that the stimulation threshold would be lower in patients with recurrent metastasis with marginal significance (*p* = 0.049). When fitting the variables collected from the highlighted case into this final model (Figure 5B), the estimated stimulation threshold was 10.22 mA with a 95% confidence interval of 6.76–13.68 mA, which is very close to the actual stimulation threshold we had in this case (i.e., 10.2 mA after normalization). Therefore, there is no evidence suggesting that the patient in the highlighted case was an outlier in our cohort with respect to the clinical presentation, stimulation parameter and intraoperative anesthesia management. The relatively high stimulation threshold reported for the patient in the highlighted case was in line with the data collected from the other 74 subjects, which was related to the patient’s specific mapping location and high BSR at the time of mapping.

## 4. Discussion

In this retrospective study, we present a unique case of interlimb motor facilitation induced by peripheral nerve stimulation in a patient with a high-grade glioma near the medial peri-Rolandic area (i.e., Zone 1), which has never been reported in the literature. With facilitation, the cMEPs were elicited at a much lower current, less than one-quarter of the mapping thresholds typically used in Zone 1. This unique finding led us to hypothesize the potential utility in exploiting this facilitation phenomenon to reduce the risks associated with excessive stimulation, such as stimulation-induced seizures, false localization due to current spread, and tissue damage. For this reason, we conducted a retrospective review study of IONM data collected from brain tumor patients at our center over the last 4 years to search for similar phenomena that may have been overlooked previously. Although it was frustrating to realize that the interlimb facilitation effect was merely coincidental and was not present in the other 74 patients who underwent motor mapping under general anesthesia, we were able to determine the facilitation effect was not related to the patient’s specific demographic information, intraoperative motor mapping paradigm or anesthesia management. We also identified variables that can impact the cortical motor mapping threshold, which provided us with a practical model to guide future cortical motor mapping procedures. Our review also stressed the volatility of stimulation-induced seizures in this patient cohort even when the BSR was over 70%, underpinning our intent to exploit cMEP facilitation effect to reduce the occurrence of stimulation-induced seizures.

When determining the optimal stimulation intensity for motor mapping, two key factors to consider are the location of stimulation and the level of burst suppression. Our model corroborated the findings from prior research investigating the effects of EEG suppression on cortical motor mapping thresholds (17). We further determined that the specific stimulation location along the motor cortex could also profoundly affect the motor mapping threshold. The hand motor region (Zone 2) was deemed to have a much lower stimulation threshold than the other areas. Simply put, we should treat the motor mapping in each zone differently. A 10-mA stimulation in Zones 1 and 3 may be acceptable, but it might be too high for mapping in Zone 2, which may indicate incorrect localization of the primary motor cortex and possibly result in stimulation-induced seizures. Our study showed great promise for utilizing statistical learning methods to predict appropriate stimulation parameters for efficient and safe cortical motor mapping.

Our extensive review of the other 74 patients also validated that demographic and clinical presentations of the patient in the highlighted case were within the typical range in this cohort. Although the stimulation current appeared to be high in the highlighted case (i.e., 17 mA with a pulse duration of 300 μs, equivalent to 10.2 mA with a pulse duration of 500 μs), our analysis with a linear regression model indicated that the high stimulation threshold was associated with the location of the stimulation (i.e., Zone 1) and the BSR (i.e., 80%) at the time of motor mapping for this patient. Using the model established based on the data collected from 74 other patients, we were able to demonstrate that the actual stimulation intensity utilized in the highlighted case fell within the 95% confidence interval of the predicted stimulation threshold. Therefore, the highlighted case was not an outlier in terms of either the stimulation intensity or any other demographic or clinical variables.

In the highlighted case, MEP facilitation was observed in upper extremity muscles innervated by cervical spinal nerve roots following stimulation of the PTN at the ankle (i.e., lumbosacral in origin). This observation carries some similarity to the findings from studies using tetanic stimulation (i.e., 50-Hz stimulation of a peripheral nerve prior to MEP stimulation) to augment the MEP amplitude. Tetanic stimulation of unilateral tibial nerve also can augment MEP amplitudes of bilateral leg muscles as well as remote muscles that are not innervated by the tibial nerve (i.e., hand muscles) at the same time (8), suggesting some mechanisms at the levels of the brain and/or spinal cord may be involved in this facilitation mechanism. The post-tetanic facilitation of the facial nerve did not yield any MEP augmentations of facial muscles and limb muscles (18). Meanwhile, tetanic stimulation of the ulnar nerve augmented MEP amplitude of bilateral hand muscles, but not the facial muscles (18). This finding may support the argument that potentiation of the corticospinal tract, instead of the corticobulbar tract, plays a crucial role in the tetanic facilitation mechanism. It also suggests that augmentation most likely occurred at the level of the spinal cord or subcortex, instead of the primary motor cortex (18). Using F-wave techniques, Yamamoto et al. (7) demonstrated that tetanic stimulation of peripheral nerves (i.e., median nerves) increases the excitation of spinal cord anterior horn cells regardless of the spinal level corresponding to the stimulated peripheral nerves, which in turn causes remote augmentation of MEPs in the lower extremities, similar to our interlimb facilitation. However, the interlimb facilitation phenomenon we observed does not resemble the post-tetanic facilitation in a few aspects. First, we stimulated the tibial nerve at a very low rate (i.e., 2.79 Hz) in comparison to the tetanic stimulation rate. It is possible that rare metastasis in the spinal cord resulting from glioblastoma progression (19) may have allowed low-frequency stimulation of peripheral nerves to increase the excitability of the anterior horn cells, but we do not have any evidence to suggest this particular patient had observable changes in the spinal cord. It should be noted that we did not stimulate the ipsilateral (right-side) PTN to see if it can also induce MEP facilitation on the contralateral (left-side) upper extremity in the highlighted case, limiting our ability to determine whether the observed interlimb facilitation phenomenon shares the same mechanism as the post-tetanic facilitation. Second, there was no facilitation effect from stimulating either the median nerve (C5-T1) or the ulnar nerve (C8-T1) in our case, individually or combined, indicating that this interlimb facilitation phenomenon is unlikely originated from the excitation of anterior horn cells in the spinal cord. In addition, the mechanisms underlying MEP augmentation by tetanic stimulation on peripheral nerves are still undetermined and controversial (10). We cannot ignore the fact that tetanic stimulation of the pudendal nerve could result in a more pronounced MEP facilitation effect than tetanic stimulation of the bilateral median nerves and unilateral tibial nerve combined (9, 10). Sasaki et al. proposed the MEP augmentation effect by tetanic stimulation may also occur at a basal ganglia and cerebral cortex level (10).

On the other hand, from a neuroanatomic perspective, the somatosensory cortex is posterior to the primary motor cortex, separated by the central sulcus. The sensorimotor areas of the upper arm and shoulder are in proximity to those areas of the lower extremity (20). Therefore, a cranial origin of facilitation appears to be more probable in this case. In addition, tumor infiltration and repeated surgical resection are two major risk factors for functional reorganization (21–24). The patient in the highlighted case had a complicated medical history, with progression from WHO grade III to grade IV glioma over a 3-year course, multiple prior surgical resections, and multiple ancillary therapies, which may all have contributed to the functional reorganization in the brain. At a microscopic level, glioma induces neuronal hyperexcitability in the microenvironment within peritumoral tissues, involving calcium-related signaling and glutamate release (25, 26). High-gamma frequency band activities have been observed in tumor-infiltrated areas in adult glioblastoma patients (26). Peritumoral neurons exhibited spontaneous and evoked epileptiform activities and were more susceptible to chemically induced hyperexcitability (25). Of note, there is also growing evidence showing that gliomas induce changes in resting-state functional connectivity not only in the adjacent cortex but also in remote non-lesional areas (27, 28). Taken together, we postulate that the interlimb cMEP facilitation observed in the highlighted case is owed to some unique glioma-related mesoscopic functional reorganization and microscopic neurophysiological changes in the brain that occurred by chance. However, we cannot rule out the possibility of spinal potentiation being involved in this facilitation phenomenon. We do not have enough measurements or data to determine the definite cause of this interlimb facilitation phenomenon.

Although the interlimb facilitation presented in the highlighted case appears to be coincidental, the prospect of cMEP facilitation remains appealing as a technique for reducing the risk of stimulation-induced seizures. Despite all the safety measures being utilized clinically, the incidence of stimulation-induced seizures ranges from 2.1 to 24.9% in the current literature (29–36). In our patient cohorts, the stimulation-induced seizure occurrence was approximately 4.05% under general anesthesia with a BSR of 60 ± 10% using a train of 500-Hz stimulation (10.73 ± 3.36 mA) for motor mapping. BSR has been intentionally induced for neuroprotection and seizure reduction (37); however, patients may still have stimulation-induced seizures even when the BSR was 70% in our patient cohort. Although we did not find that the stimulation thresholds for inducing seizures were significantly higher than the motor mapping thresholds, stimulation thresholds were all above the 60th quantile of motor mapping thresholds in the corresponding zones. It is evident that a high stimulation current may lead to stimulation-induced seizures, which is not easily predictable.

In addition, in this study, stimulation-induced seizures were all observed in patients with glioma, two with high-grade glioma and one with low-grade glioma. Over 80% of patients with low-grade gliomas and 40–60% of glioblastoma patients present with seizures and have a concomitant epilepsy diagnosis (38, 39). Glioma-related seizures and neuronal hyperexcitability are closely associated with the progression and regrowth of gliomas (25, 26, 40). Our cohort of patients with brain metastases did not have any seizures intraoperatively, however there is still a risk of perioperative seizures in this patient population. In fact, nearly 11% of patients with brain metastases develop seizures preoperatively (41), and the odds of a stimulation-induced seizure intraoperatively are similar to those in glioma patients (29). Overall, a history of preoperative seizures can significantly increase the occurrence of intraoperative stimulation-induced seizures (42). Moreover, intraoperative stimulation-induced seizures have a negative impact on postoperative outcomes with higher complication rates (43). Therefore, we should limit the occurrence of stimulation-induced seizures at all costs. Minimizing the stimulation intensity seems to be the most straightforward way to reduce seizure occurrence, which may also help prevent tissue damage and potential tumor progression from excessive stimulation. The potential for facilitation techniques to reduce stimulation intensities was what truly motivated the investigation into this intriguing cortical mapping case and the subsequent retrospective review. It also underlined the need for a better understanding of the mechanisms of cMEP facilitation techniques, which will be crucial for reliable and consistent monitoring of the motor pathway.

There were several limitations to this retrospective review study. First, this study had a limited sample size. Our medical center, located in rural New Hampshire, serves a population of 1.9 million in northern New England. Our patient cohort is relatively small compared to larger urban health systems and does not present substantial racial or ethnic diversity. We were unable to obtain sufficient power to analyze data related to detailed medical histories (i.e., baseline neurological disorders), pathological features or individual tumor morphologies and characteristics. Second, as this was only a retrospective review study, we did not prospectively design the study to include other established facilitation techniques (i.e., post-tetanic stimulation) in cMEP monitoring, which will require separate approval by the IRB. A third limitation was related to the linear regression model that we used to infer the relationship between stimulation currents and certain clinical variables. There are some factors that we believe can significantly affect the model fitting, but we were not able to include them in the current model as any quantitative or categorical variables. For example, we did not consider the precision of the motor mapping technique utilized in this study and only grouped the mapping data into different zones. Motor mapping was performed through the strip electrodes with an interelectrode distance of 10 mm and an exposed electrode diameter of 5 mm. The placement of the strip electrodes was subject to the surgeon’s access and experience. We did not determine or document the specific stimulation location (i.e., x, y, z coordinates) on the motor cortex for each patient, which theoretically can be done *via* the neuro-navigation system. However, the latest findings at our institution have demonstrated that neuro-navigation based on preoperative radiological images could have an error up to 17 mm just by opening the dura matter during craniotomy due to the brain shift and deformation related to the tumor (44, 45). These constraints render it impossible to identify the absolute location on the primary motor cortex that triggers cMEPs with the lowest current threshold in a fast-moving operating room environment. Other omitted factors related to the tumor’s heterogeneous nature, such as its dimension, relative distance to the motor or sensory area, and mass effect on the motor tract, could also affect the motor mapping threshold as well.

These limitations highlight the need for further research on the effects of facilitation and glioma-induced meso-and microscopic functional changes on intraoperative motor mapping. The interlimb facilitation effect presented in this manuscript likely had contribution from cortical reorganization both structurally and functionally, which unexpectedly enabled us to use a much lower stimulation intensity to provide continuous motor monitoring during tumor resection. Even though the likelihood of reproducing such phenomena in other patients is low, adopting the post-tetanic facilitation technique in cortical motor mapping may reduce the stimulation current needed to elicit cMEPs and, hence, lower the risks of triggering seizures and inducing tissue damage or even tumor regrowth. However, it remains to be evaluated whether facilitation would compromise the reliability of cMEP monitoring, i.e., making it less sensitive for detecting iatrogenic injuries to the motor pathway. The reliability of utilizing facilitation techniques in brain tumor patients could be questionable as well, as the tumor may have invaded or infiltrated within the crucial structures potentially involved in the facilitation mechanism.

## 5. Conclusion

In summary, this retrospective study reports a unique finding of interlimb cMEP facilitation in a patient with a recurrent peri-Rolandic high-grade glioblastoma who underwent surgical resection. With the facilitation effect, the cMEP stimulation threshold was reduced from 17 to 11 mA in this highlighted case, which may have the benefits of reducing simulation-induced seizure occurrence and preventing tissue damage. However, even though 64.86% of the reviewed cases had documented intraoperative cMEP changes, we did not identify the abovementioned facilitation phenomenon in 74 other patients with similar clinical presentations, intraoperative motor mapping paradigms and anesthesia management. We further determined that the stimulation location on the motor cortex and the BSR in the ECoG data could significantly affect the motor mapping stimulation threshold. The relatively high stimulation threshold (i.e., 17 mA) utilized in the highlighted case was mostly due to its mapping location (i.e., Zone 1) and BSR (i.e., 80%), which is in accordance with the data collected from the other 74 patients. Our findings corroborated prior research endeavors regarding the effects of EEG suppression on cortical motor mapping thresholds, showed promise for utilizing statistical learning methods to predict appropriate stimulation parameters, and provided a practical guide to cortical stimulation in brain tumor patients under general anesthesia. Although these data are unlikely to reveal the definite underlying mechanism of this facilitation effect, we suspect this effect was a result of unique functional/structural reorganization caused by glioma progression and multiple prior resections in this specific patient. Further research needs to be conducted to determine the clinical benefits and relevance of utilizing other established facilitation techniques in intraoperative motor mapping during craniotomies.

## Data availability statement

The original contributions presented in the study are included in the article/Supplementary material, further inquiries can be directed to the corresponding author.

## Ethics statement

The studies involving human participants were reviewed and approved by the Human Research Protection Program, Dartmouth-Hitchcock Medical Center. Written informed consent for participation was not required for this study in accordance with the national legislation and the institutional requirements.

## Author contributions

YS, LE, and EK contributed to conception and design of the study. YS and ZL organized the database. YS and PM-C performed the statistical analysis. YS, JS, JK, and WS wrote the first draft of the manuscript. ZL, MDB, and AA wrote sections of the manuscript. All authors contributed to the manuscript revision, read, and approved the submitted version.

## Conflict of interest

The authors declare that the research was conducted in the absence of any commercial or financial relationships that could be construed as a potential conflict of interest.

## Publisher’s note

All claims expressed in this article are solely those of the authors and do not necessarily represent those of their affiliated organizations, or those of the publisher, the editors and the reviewers. Any product that may be evaluated in this article, or claim that may be made by its manufacturer, is not guaranteed or endorsed by the publisher.
