# Supplementary material for: Variables associated with cortical motor mapping thresholds: A retrospective data review with a unique case of interlimb motor facilitation
Source: Front Neurol. 2023 Apr 11;14:1150670. doi: 10.3389/fneur.2023.1150670 (PMC10128911; doi:10.3389/fneur.2023.1150670)
Supplement: Supplementary file 1 [file Data_Sheet_1.docx]

# **Supplemental material**

## **Anesthesia**

All patients were induced under general endotracheal anesthesia. Methods of induction and use of paralytics were at the discretion of attending anesthesiologists. After induction, patients were transitioned to a total intravenous anesthetic (TIVA) with a combination of propofol and remifentanil infusion for the remainder of the procedure.

## **Surgical Approach**

Patients were positioned supine, endotracheally intubated, and placed in a three-point fixation Mayfield clamp with the head in neutral alignment. Neuro-navigation (Stealth Station S8, Medtronic Inc., Dublin, Ireland) was used, and registration with preoperative MRI was performed. Subsequently, patients’ hair was clipped over the planned incision site, and the area was prepped and draped using a sterile technique.

A scalp incision was made, and a skin flap was reflected to expose the cranium. Neuro-navigation was then used to confirm the location of the sagittal sinus and the extent of craniotomy required to encompass the tumor. Craniotomy was performed with complete exposure of the sagittal sinus. Sharp dural opening was performed to expose the tumor and surrounding cortical surface.

Ultrasound (BK5000, BK Medical, Peabody, MA) was used to identify the tumor location. The surgical microscope was used, and 1x4 subdural strip electrodes were placed on top of the cortical surface. The IONM protocol was performed as described below to localize the central sulcus and establish recording locations for SSEP and MEP monitoring. Tumor dissection proceeded under the surgical microscope with intraoperative SSEP and MEP monitoring intermittently performed to guide the resection. Once maximal safe resection was achieved based on IONM and neuronavigation, the dura was reapproximated, and the bone returned to its native position and then was secured with titanium implants. The scalp was subsequently closed in a layered fashion, and patients were awakened from anesthesia and extubated.

## **Intraoperative Neuromonitoring**

Following general anesthesia and prior to surgical incision, subdermal needle electrodes were placed on the contralateral side of the face (orbicularis oculi, orbicularis oris, and tongue), upper extremity (trapezius, deltoid, biceps, triceps, extensor carpi radialis, abductor digiti minimi, first dorsal interosseous, and abductor pollicis brevis), and lower extremity (adductor longus, vastus lateralis, gastrocnemius, tibialis anterior, and abductor hallucis brevis). Subdermal needle electrodes were also placed over the contralateral facial, ulnar, median, and posterior tibial nerves. A reference electrode for recording ECoG signals and functional mapping was placed on the scalp, distant from the ipsilateral primary motor and sensory areas. After reflection of the dural flap, a 1x4 subdural strip electrode (MS04R-IP10X-0JH, ADtech, Oak Creek, WI) was placed by the surgeon over the cortical surface adjacent to the tumor to record ECoG signals as well as to perform functional mapping. In some cases, the surgeon may have placed two 1x4 or one 2x4 strip electrodes to cover both medial and lateral primary motor and sensory areas to facilitate monitoring the sensorimotor functions of both upper and lower extremities.

The phase reversal pattern induced by median nerve stimulation has been utilized as an important feature during IONM to accurately identify the hand sensorimotor areas and central sulcus. Cortical SSEPs with nearly mirror-image waveforms (i.e., opposite polarities) can be recorded by electrodes covering the hand sensorimotor areas anterior to the central sulcus, with a negative peak at the sensory cortex and a positive peak at the motor cortex. However, in the medial peri-Rolandic area, it is almost impossible to obtain a reliable phase reversal pattern by stimulating the median nerve or posterior tibial nerve. Therefore, reliably localizing the primary motor, sensory areas and central sulcus in the medial peri-Rolandic area poses a challenge when the lateral aspect of the sensorimotor regions are outside the cortical exposure.

To map motor function, a high-frequency (i.e., 500 Hz) multipulse (7-8 pulses, pulse duration = 0.3 or 0.5 ms) monopolar stimulus was delivered to the electrode covering the motor cortex. The anodal stimulation commenced at 1 mA and increased in 1-mA increments until MEPs were elicited in monitored muscles, after-discharges or seizures were triggered, or 20 mA was attained. Cathodal stimulation was also attempted to see if any MEPs could be reliably elicited at a lower stimulation threshold. Each electrode contact covering the motor cortex, as determined by phase reversal technique, was stimulated. When phase reversal technique was not informative in the medial peri-Rolandic area, all electrode contacts were stimulated to map the motor cortex. We picked the electrode contact that needed the lowest stimulation current to elicit MEPs for continuous motor monitoring. A ball-tip probe was utilized to map both the cortical and subcortical motor pathways. Upper and lower limb SSEPs were acquired interleaved with cortical MEPs throughout the monitoring period. ECoG signals were continuously monitored by a board-certified clinical neurophysiologist to identify after-discharges or seizures.

# **Supplemental figure legends**

**Supplemental Figure 1. Potential facilitation effect from posterior tibial nerve stimulation.** 69-year-old femaile patient presented with a metastatic carcinoma in the right frontal parafalcine area. We performed intraoperative motor and sensory mapping during the tumor resection. **(A)** Pre-operative MR images in coronal and sagittal views, with a red arrow pointing towards the tumor. **(B)** Cortical motor evoked potential (cMEP) of the left-side lower extremity muscles. cMEPs were elicited by stimulating the right medial motor cortex at 11 mA. Posterior tibial nerve (PTN) was stimulated for 5-10 seconds before the cMEP stimulation. We noticed an increase in the amplitude of cMEP on abductor hallucis brevis (AH) muscle, when the PTN stimulation was increased gradually from 20 mA to 40 mA. However, this effect was not consistent. ADD: adductor longus, QD: quadriceps, TA: tibialis anterior, GST: gastrocnemius.
