# Supplementary figures and images for: Variables associated with cortical motor mapping thresholds: A retrospective data review with a unique case of interlimb motor facilitation
Source: Front Neurol. 2023 Apr 11;14:1150670. doi: 10.3389/fneur.2023.1150670 (PMC10128911; doi:10.3389/fneur.2023.1150670)

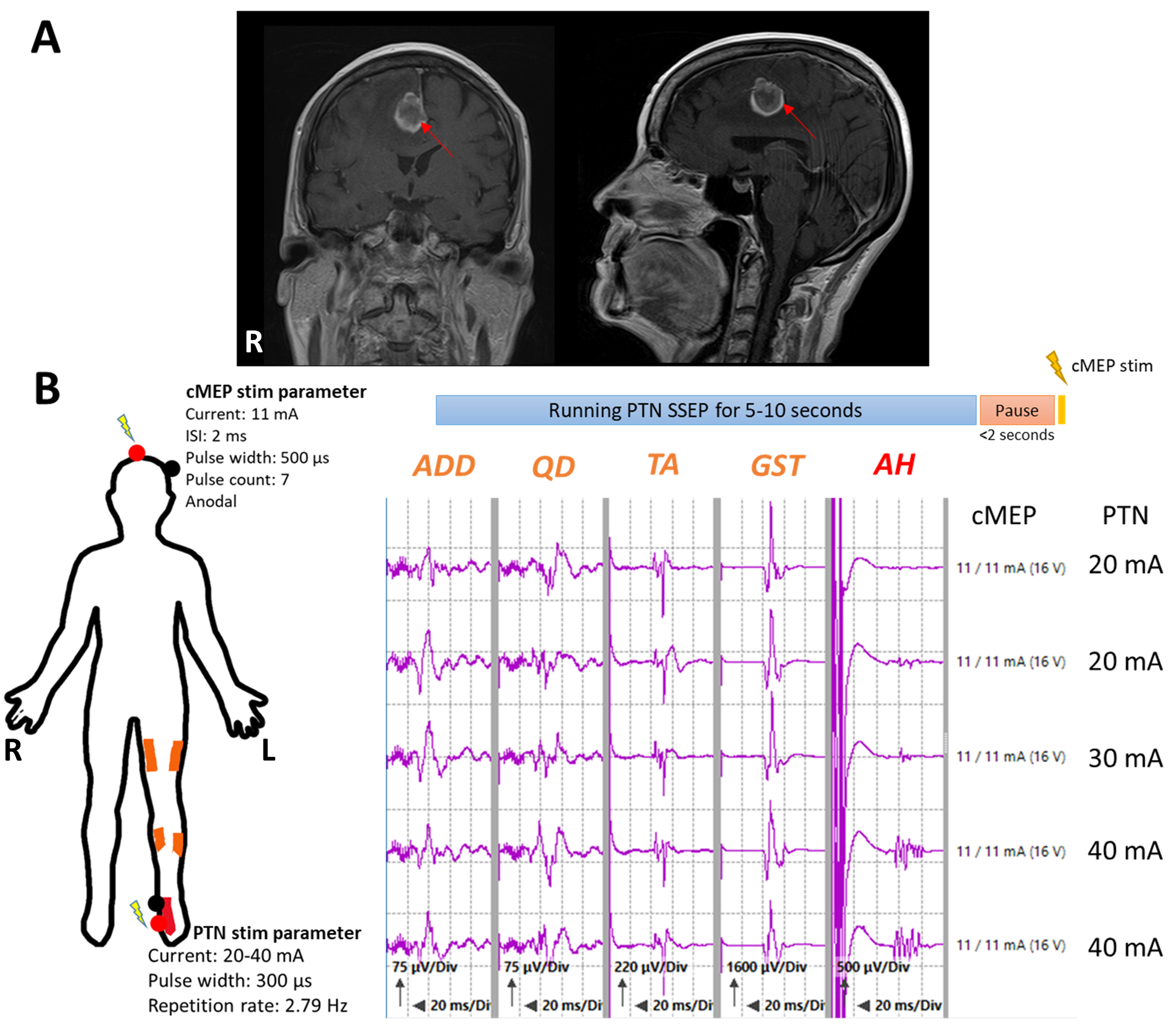

Supplement: Supplementary file 2 [file Image_1.TIF]
